# Supplementary material for: Evaluation of quality of life and associated factors in patients with intertrochanteric femoral fracture
Source: PLoS One. 2023 Nov 21;18(11):e0293686. doi: 10.1371/journal.pone.0293686 (PMC10662741; doi:10.1371/journal.pone.0293686)
Supplement: Attachment — Submitted filename: PONE-D-23-23806 edits.pdf [file pone.0293686.s003.pdf]

PLOS ONE

Evaluation of quality of life and associated factors in patients with intertrochanteric femoral fracture

--Manuscript Draft--

**Manuscript Number:**	PONE-D-23-23806
**Article Type:**	Research Article
**Full Title:**	Evaluation of quality of life and associated factors in patients with intertrochanteric femoral fracture
**Short Title:**	Quality of Life after Intertrochanteric Fracture
**Corresponding Author:**	Mehdi Tavasoli, M.D<br>Babol University of Medical Science Faculty of Medicine<br>Babol, IRAN, ISLAMIC REPUBLIC OF
**Keywords:**	Quality of Life, Intertrochanteric Fractures, Outcome, Follow-Up, Iran.
**Abstract:**	Background: Intertrochanteric fracture is a common injury among the elderly population causing fundamental lifestyle derangements, which influences the patients' social and psychological functioning. We aimed to study the quality of life (QoL) and its different parameters in patients with this type of injury.<br>Materials and methods: In this cohort study, all patients hospitalized with intertrochanteric fracture and aged above 50 from 2020 to the 2022 at the Shahid Beheshti Hospital in Babol, Iran, were included. Patients were managed mostly surgically and in exceptional cases non-surgically, were followed up for at least 12 months after receiving treatment. During the follow-up period, patients were dialed and completed a questionnaire to assess the patients' QoL by the 36-item Short Form Health Survey (SF-36). QoL parameters were analyzed based on patients' sex, age, type of treatment, and height of fall causing fracture.<br>Result: A total number of 200 patients including 101 (50.5%) males and 99 (49.5%) females with mean age of patients was 74.76 ± 11.36 years (range: 50-99) were included. Regarding the received treatment, 192 (96.0%) patients underwent surgery, and 8 (4.0%) underwent non-surgical treatment. In the study of SF-36 scores, the mean score of male patients was 42.31±14.58 and females scored 37.83±15.35, and the difference was statistically significant (P=0.04). The mean score of QoL and its subscales among the patients of the 50-75 group was significantly higher than the 76-99 group (P<0.001). The average score of QoL was considerably higher in patients who had surgery (40.75±14.57) compared to those who has non-surgical treatment (24.30±19.85) (P=0.01). Patients having a fall from a higher height had higher QoL after treatment.<br>Conclusion: This study revealed that patients with an intertrochanteric femoral fracture had a poor QoL in all aspects. The overall QoL was significantly higher among male patients, younger patients, those who underwent surgical treatment, and the falls from higher heights. These findings highlight the necessity of long-term follow-up and support in patients with intertrochanteric fractures.
**Order of Authors:**	Sina Afzal, M.D
	Seyyed Mokhtar Esmaeilnejad-Ganji, Professor
	Fatemeh Ghasemi, M.D
	Mojtaba Baroutkoub, assistant professor
	Hasan Barati, assistant professor
	Mehdi Tavasoli, M.D
**Additional Information:**	
**Question**	**Response**
**Financial Disclosure**	The author(s) received no specific funding for this work.

Powered by Editorial Manager® and ProduXion Manager® from Aries Systems Corporation

<table>
<tr><td>
Enter a financial disclosure statement that describes the sources of funding for the work included in this submission. Review the submission guidelines for detailed requirements. View published research articles from PLOS ONE for specific examples.

This statement is required for submission and will appear in the published article if the submission is accepted. Please make sure it is accurate.

Unfunded studies
Enter: The author(s) received no specific funding for this work.

Funded studies
Enter a statement with the following details:
• Initials of the authors who received each award
• Grant numbers awarded to each author
• The full name of each funder
• URL of each funder website
• Did the sponsors or funders play any role in the study design, data collection and analysis, decision to publish, or preparation of the manuscript?
• NO - Include this sentence at the end of your statement: The funders had no role in study design, data collection and analysis, decision to publish, or preparation of the manuscript.
• YES - Specify the role(s) played.

* typeset
</td><td></td></tr>
<tr><td>
Competing Interests

Use the instructions below to enter a competing interest statement for this submission. On behalf of all authors, disclose any competing interests that could be perceived to bias this work—acknowledging all financial support and any other relevant financial or non-financial competing interests.

This statement is required for submission and will appear in the published article if the submission is accepted. Please make
</td><td>The authors have declared that no competing interests exist.</td></tr>
</table>

sure it is accurate and that any funding sources listed in your Funding Information later in the submission form are also declared in your Financial Disclosure statement.<br><br>View published research articles from *PLOS ONE* for specific examples.<br><br>**NO authors have competing interests**<br><br>Enter: *The authors have declared that no competing interests exist.*<br><br>**Authors with competing interests**<br><br>Enter competing interest details beginning with this statement:<br><br>*I have read the journal's policy and the authors of this manuscript have the following competing interests: [insert competing interests here]*<br><br>* typeset	
**Ethics Statement**<br><br>Enter an ethics statement for this submission. This statement is required if the study involved:<br><br>• Human participants<br>• Human specimens or tissue<br>• Vertebrate animals or cephalopods<br>• Vertebrate embryos or tissues<br>• Field research<br><br>Write "N/A" if the submission does not require an ethics statement.<br><br>General guidance is provided below. Consult the submission guidelines for detailed instructions. **Make sure that all information entered here is included in the Methods section of the manuscript.**	This study was conducted per the Declaration of Helsinki. All patients' data have been anonymized prior to analysis. All procedures were performed in accordance with the Declaration of Helsinki and were approved by the research ethics committee of School of Medicine at Babol University of Medical Sciences, Babol, Iran (code: IR.MUBABOL.REC.1399.007). All patients provided written informed consent to participate in the study and for the publication of its results.

Powered by Editorial Manager® and ProduXion Manager® from Aries Systems Corporation

**Format for specific study types**<br><br>**Human Subject Research (involving human participants and/or tissue)**<br>• Give the name of the institutional review board or ethics committee that approved the study<br>• Include the approval number and/or a statement indicating approval of this research<br>• Indicate the form of consent obtained (written/oral) or the reason that consent was not obtained (e.g. the data were analyzed anonymously)<br><br>**Animal Research (involving vertebrate animals, embryos or tissues)**<br>• Provide the name of the Institutional Animal Care and Use Committee (IACUC) or other relevant ethics board that reviewed the study protocol, and indicate whether they approved this research or granted a formal waiver of ethical approval<br>• Include an approval number if one was obtained<br>• If the study involved *non-human primates*, add *additional details* about animal welfare and steps taken to ameliorate suffering<br>• If anesthesia, euthanasia, or any kind of animal sacrifice is part of the study, include briefly which substances and/or methods were applied<br><br>**Field Research**<br><br>Include the following details if this study involves the collection of plant, animal, or other materials from a natural setting:<br>• Field permit number<br>• Name of the institution or relevant body that granted permission	
**Data Availability**<br><br>Authors are required to make all data underlying the findings described fully available, without restriction, and from the time of publication. PLOS allows rare exceptions to address legal and ethical concerns. See the PLOS Data Policy and FAQ for detailed information.	Yes - all data are fully available without restriction

A Data Availability Statement describing where the data can be found is required at submission. Your answers to this question constitute the Data Availability Statement and **will be published in the article**, if accepted.<br><br>**Important:** Stating 'data available on request from the author' is not sufficient. If your data are only available upon request, select 'No' for the first question and explain your exceptional situation in the text box.<br><br>Do the authors confirm that all data underlying the findings described in their manuscript are fully available without restriction?	
**Describe where the data may be found in full sentences. If you are copying our sample text, replace any instances of XXX with the appropriate details.**<br><br>• If the data are **held or will be held in a public repository**, include URLs, accession numbers or DOIs. If this information will only be available after acceptance, indicate this by ticking the box below. For example: *All XXX files are available from the XXX database (accession number(s) XXX, XXX.*).<br>• If the data are all contained **within the manuscript and/or Supporting Information files**, enter the following: *All relevant data are within the manuscript and its Supporting Information files.*<br>• If neither of these applies but you are able to provide **details of access elsewhere**, with or without limitations, please do so. For example:<br><br>*Data cannot be shared publicly because of [XXX]. Data are available from the XXX Institutional Data Access / Ethics Committee (contact via XXX) for researchers who meet the criteria for access to confidential data.*<br><br>*The data underlying the results presented in the study are available from (include the name of the third party*	The data will be available for sharing by the corresponding author based on a reasonable request.

Powered by Editorial Manager® and ProduXion Manager® from Aries Systems Corporation

and contact information or URL).<br>• This text is appropriate if the data are owned by a third party and authors do not have permission to share the data.<br><br>* typeset	
Additional data availability information:	

Manuscript

Click here to access/download;Manuscript;Intertrochanteric draft.docx

Evaluation of quality of life and associated factors in patients with intertrochanteric femoral fracture

Short Title:

Quality of Life after Intertrochanteric Fracture

Sina Afzal [1], Seyyed Mokhtar Esmaeilnejad-Ganji[2], Fatemeh Ghasemi[2], Mojtaba baroutkoub[1], Hasan Barati[1], Mehdi Tavassoli[2*]

1. Department of Orthopedic and Trauma Surgery, Shahid Beheshti University of Medical Sciences, Tehran, Iran.

2. Department of Orthopedic and Trauma Surgery, Babol University of Medical Sciences, Babol, Iran.

*** Correspond Author:**
Mehdi Tavassoli
ORCID: 0000-0001-9509-1723
Mehdi.tavasolii@gmail.com

First Author:

Sina Afzal
ORCID: 0009-0000-6666-9207

Sina.afzal@gmail.com

Abstract

Background: Intertrochanteric fracture is a common injury among the elderly population causing fundamental lifestyle derangements, which influences the patients' social and psychological functioning. We aimed to study the quality of life (QoL) and its different parameters in patients with this type of injury.

Materials and methods: In this cohort study, all patients hospitalized with intertrochanteric fracture and aged above 50 from 2020 to the 2022 at the Shahid Beheshti Hospital in Babol, Iran, were included. Patients were managed mostly surgically and in exceptional cases non-surgically, were followed up for at least 12 months after receiving treatment. During the follow-up period, patients were dialed and completed a questionnaire to assess the patients' QoL by the 36-item Short Form Health Survey (SF-36). QoL parameters were analyzed based on patients' sex, age, type of treatment, and height of fall causing fracture.

Result: A total number of 200 patients including 101 (50.5%) males and 99 (49.5%) females with mean age of patients was 74.76 ± 11.36 years (range: 50-99) were included. Regarding the received treatment, 192 (96.0%) patients underwent surgery, and 8 (4.0%) underwent non-surgical treatment. In the study of SF-36 scores, the mean score of male patients was 42.31±14.58 and females scored 37.83±15.35, and the difference was statistically significant ($P=0.04$). The mean score of QoL and its subscales among the patients of the 50-75 group was significantly higher than the 76-99 group ($P<0.001$). The average score of QoL was considerably higher in patients who had surgery (40.75±14.57) compared to those who has non-surgical treatment (24.30±19.85) ($P=0.01$). Patients having a fall from a higher height had higher QoL after treatment.

Conclusion: This study revealed that patients with an intertrochanteric femoral fracture had a poor QoL in all aspects. The overall QoL was significantly higher among male patients, younger patients, those who underwent surgical treatment, and the falls from higher heights. These

findings highlight the necessity of long-term follow-up and support in patients with intertrochanteric fractures.

Keywords: Quality of Life, Intertrochanteric Fractures, Outcome, Follow-Up, Iran.

Introduction

Intertrochanteric fracture is a form of femoral fracture involving both the lesser and greater femoral trochanters and is a common injury among the elderly population (1). In this kind of the proximal femoral fracture, a fracture line travels obliquely through the trochanters below the femoral neck, and does not involve the neck or upper components (2). Intertrochanteric fractures can develop in the elderly as a result of minor trauma like falling or in younger population as a result of severe trauma like high-energy motor vehicle accident (3). Incidence rate of the intertrochanteric fractures is estimated about 171 per 100,000 population in the United States that bears nearly 52500 USD economic burden for each patient (4). Women are more likely to suffer from this form of fracture, which is thought to be due to metabolic and hormonal variations in the bone (5). Studies from Iran have reported mortality rates about 30-35% among patients with intertrochanteric fracture who underwent surgical treatment (6, 7).

In the physical examination, the injured limb is generally shortened, the region is inflamed, and movement of the limb is painful (8). Surgical treatment is the current gold standard therapy for intertrochanteric fractures (9). Non-surgical treatment, on the other hand, offer a satisfactory rate of improvement (8). Malignancy, failure of the operated device such as protrusion of the device or sinking of the rod in the hip joint, shortening of the limbs, nonunion, and in rare cases avascular necrosis of the femoral head could be associated with intertrochanteric fractures (10, 11). Intertrochanteric fractures cause a lot of morbidity and mortality, so knowing about postoperative complications can help patients have a better quality of life (QoL) (7).

Today, there is a significant focus on the QoL of patients with trauma and fractures since evidence shows that the QoL following trauma is much lower than before the event (11). Thus, patients should be followed up after receiving surgical treatment for intertrochanteric fracture since the

altered QoL still remains low (6, 12). Patients' poor QoL after surgery may be due to the lack of follow-up after discharge, extensive medical expenditures, inadequate rehabilitation, or a lack of social, physical, and emotional support (13, 14, 15). Therefore, in this study we aimed to assess the QoL in patients undergoing surgery for intertrochanteric fracture to help clinicians in improving the care provided for such patients with a final goal of enhancing surgical and patient-reported outcomes.

Material and methods

Study design and population

In this cohort study, all patients hospitalized with intertrochanteric fracture from 07/04/2020 to the 07/04/2022 at the Shahid Beheshti Hospital in Babol, Iran, were included. Inclusion criteria were having intertrochanteric fracture and aged above 50 at the time of hospitalization or surgery. Exclusion criteria from study were the duration between fracture and admission to the hospital being more than three weeks, established dementia, or if the cause of fracture was car accident or fall from a height of more than two meters, and patients with a severe underlying condition.

Patient management

The patient's unsatisfactory general state, the inability of anesthesia, the delay in treatment (for more than three weeks from the time of fracture), and the patient's preference to not go under surgery were all reasons for non-surgical treatment. Otherwise, the patients were treated surgically. Regarding the patients who underwent the surgical management, on average 3-5 days after surgery, patients were discharged with physiotherapy training and appropriate recommendations, while non-surgical patients were discharged after the necessary training, cane preparation, or stretching to continue treatment at home. During hospitalization, these therapies lasted an average of at least three days. Patients were contacted at least 12 months after being admitted to the hospital or having surgery.

Data collection and follow up

During the follow-up period, patients were dialed and completed a questionnaire to assess the patients' QoL by the 36-item Short Form Health Survey (SF-36). This questionnaire contains 36 questions and eight subscales, each with two to ten items. The eight subscales of this questionnaire are: physical function (PF), role disorder due to physical health (RP), role disorder due to emotional health (RE), energy/fatigue (EF), emotional well-being (EW), social function (SF), Pain (P) and general health (GH) (16). In this questionnaire, a lower score indicates a lower quality of life and vice versa. Ala, SF-36 tool has been previously translated and validated for Iranian patients and we used that version (17). QoL parameters were analyzed based on patients' sex (male/female), age group (50-75 and 76-99 years-old), type of treatment (surgical/non-surgical), and height of fall causing fracture (level height/1 meter height/2 meters height).

Statistical analysis

Categorical variables were summarized in frequency and percentage, and the quantitative variables were described by mean and standard deviation or median and interquartile range based on normality. To evaluate the statistical associations between variables, parametric (T-test) and non-parametric (Mann-Whitney and Kruskal-Wallis) tests were used based on normality assumptions of variables. SPSS version 25 was used for all data analysis. Two-sided P-values less than 0.05 were investigated and set as the level of statistical significance.

Ethical Considerations

This study was conducted per the Declaration of Helsinki. All patients' data have been anonymized prior to analysis [illegible] procedures were performed in accordance with the Declaration of Helsinki and were approved by the research ethics committee of School of Medicine at Babol University of Medical Sciences, Babol, Iran (code: IR.MUBABOL.REC.1399.007). All patients provided written informed consent to participate in the study and for the publication of its results.

Results

A total number of 200 patients including 101 (50.5%) males and 99 (49.5%) females were included in this study. The mean age of patients was 74.76 ± 11.36 years (range: 50-99). Ninety-four (47.0%) patients were in the 50-75 years age range, and 106 (53.0%) were in the age group of 76-99 years age range. Inspecting the height leading to fall and intertrochanteric fracture, 157 (78.5%) patients fell from the same height, 35 (17.5%) fell from a height of about two meters, and 8 (4.0%) fell from a height of one meter. Regarding the received treatment, 192 (96.0%) patients underwent surgery, and 8 (4.0%) patients underwent non-surgical treatment for various reasons.

In the study of SF-36 scores, the mean score of male patients was 42.31±14.58 and females scored 37.83±15.35, and the difference was statistically significant (P=0.04). However, in QoL subscales, no significant differences were observed between male and female patients with intertrochanteric fracture (Table 1).

Table 1. Comparison of quality of life subscales in male and female patients with intertrochanteric fracture.

Quality of Life	Male (mean±SD)	Female (mean±SD)	P-value
Physical function	22.52±13.74	19.39±10.76	0.22
Role disorder due to physical health	26.73±27.44	22.72±25.02	0.33
Role disorder due to emotional health	56.43±39.08	45.45±41.36	0.06
Energy / Fatigue	48.86±10.31	44.94±12.64	0.09
Emotional well-being	55.56±10.44	52.24±13.10	0.06
Social function	35.27±19.95	35.95±21.68	0.38
Pain	58.88±23.48	54.39±24.39	0.22
General health	34.20±14.96	30.55±15.46	0.07
Physical health	35.58±16.16	31.76±15.34	0.11
Mental health	49.03±15.74	43.90±17.74	0.06
Total	42.31±14.58	37.83±15.35	0.04

In the study of subscales of QoL of patients in the age groups of 50-75 and 76-99, it was found that the mean score of QoL and its subscales among the patients of the 50-75 group was

significantly higher than the 76-99 group, as the mean total score of the first group was 46.70±11.79 compared to the second group which was 34.23±15.33 (P<0.001) (Table 2).

Table 2. Comparison of quality of life subscales in patients with intertrochanteric fracture by age group.

**Quality of Life**	**50-75 age group (mean±SD)**	**76-99 age group (mean±SD)**	**P-value**
Physical function	26.70±9.26	15.86±12.70	<0.001
Role disorder due to physical health	34.84±25.45	15.80±23.73	<0.001
Role disorder due to emotional health	58.86±36.06	44.02±43.04	0.005
Energy / Fatigue	51.01±10.06	43.30±11.82	<0.001
Emotional well-being	57.95±9.84	50.33±12.48	<0.001
Social function	40.15±19.56	28.77±20.49	<0.001
Pain	65.29±23.71	49.00±21.59	<0.001
General health	38.82±12.19	26.69±15.53	<0.001
Physical health	41.41±13.18	25.58±14.86	<0.001
Mental health	51.99±13.36	41.61±18.25	<0.001
Total	46.70±11.79	34.23±15.33	<0.001

The average score of QoL was considerably higher in patients who had surgery (40.75±14.57) compared to those who has non-surgical treatment (24.30±19.85), reflecting a statistically significant difference (P=0.01), with similar significant patterns in subscales except for the two subscales of social function and general (Table 3).

Table 3. Comparison of quality of life subscales in patients with intertrochanteric fracture based on type of treatment.

**Quality of Life**	**Surgical Treatment (mean±SD)**	**Non-surgical Treatment (mean±SD)**	**P-value**
Physical function	21.14±11.37	16.87±29.02	0.03
Role disorder due to physical health	25.52±26.33	6.25±17.67	0.03
Role disorder due to emotional health	52.43±40.28	16.66±30.86	0.01
Energy / Fatigue	47.57±10.98	31.25±16.85	0.004
Emotional well-being	54.54±11.19	39.00±18.85	0.01
Social function	34.57±20.51	23.43±26.25	0.11
Pain	57.42±23.87	38.43±20.17	0.02
General health	32.81±14.99	22.50±19.82	0.11

Physical health	34.22±15.49	21.01±19.82	0.01
Mental health	47.28±16.33	27.58±20.74	0.01
Total	40.75±14.57	24.30±19.85	0.01

Based on the height of fall causing intertrochanteric, the total QoL score and subscales varied in three categories of height among the included sample of patients in this study (Table 4).

Table 4. A comparison of quality of life subscales in patients with an intertrochanteric fracture based on the height of the fall that caused fracture.

**Quality of Life**	**Level height (mean±SD)**	**Height 1 meter (mean±SD)**	**Height 2 meters (mean±SD)**	**P-value**
Physical function	19.39±12.06^{a*}	26.00±11.74^{b}	30.00±14.14^{bc}	0.001
Role disorder due to physical health	21.49±25.39^{a}	28.57±27.34^{b}	38.12±20.86^{ba}	0.003
Role disorder due to emotional health	49.46±41.27	56.19±35.94	58.33±46.29	0.58
Energy / Fatigue	46.21±11.34^{a}	50.85±11.57^{b}	51.37±12.37^{ab}	0.006
Emotional well-being	52.71±12.10^{a}	54.31±8.70^{b}	59.00±15.11^{ab}	0.005
Social function	33.51±20.94	30.14±20.89	32.81±18.82	0.55
Pain	54.69±24.18	64.00±22.53	63.12±21.07	0.09
General health	31.56±15.00	31.42±16.02	36.25±16.63	0.21
Physical health	31.78±15.62^{a}	38.25±14.74^{b}	41.12±15.58^{ab}	0.004
Mental health	45.47±17.11	51.12±15.31	46.13±18.44	0.27
Total	38.63±15.00^{a}	46.18±13.80^{b}	48.12±15.43^{ab}	0.03

*Different letters in each row indicate a significant difference between the two groups at the level of α = 0.05 using Tukey test.

Discussion

This study investigated the QoL and its different parameters among patients with intertrochanteric fracture who most of them underwent surgical treatment to fix the injury. The main findings derived from the [illegible]uited sample of patients in this study were overall significantly higher QoL among male patients with this kind of fracture, younger patients, those who underwent surgical treatment, and the falls from higher heights. Variations in subscales of QoL

was also considerable in different stratifications of patients investigated in this study reflecting varying aspects of QoL affected by such injuries.

The most notable finding of this study was those intertrochanteric femoral fractures cause derangements in patient QoL, even when there were no complications and in cases with full healing achieving. The QoL of trauma patients and those with fractures and bone and soft tissue injuries has not been well studies in Iran, and most of the available studies are from the Western countries (18). However, regarding intertrochanteric fractures, there were some studies from Iran that assesses the outcomes of treatment of these injuries and QoL of patients having the injury (6, 7, 15). In a study in Iran on 385 patients aged≥60 years-old who had intertrochanteric fracture and were treated surgically by open reduction and internal fixation by dynamic hip screw, the mortality rate after surgery was 36.9% with higher figures among males (54.9%) compared to females (41.9%), and only 33.5% of the patients had good score of QoL based on the Modified Harris Hip Score, concluding that QoL even postoperatively remains low in this patients and needs appropriate follow up to improve the treatment outcomes and patient-reported QoL (7). In another study on 110 patients with intertrochanteric fracture from Iran who were treated by dynamic hip screw and the QoL was assessed by the SF-36 similar to our study, reported no association between the overall score of QoL and its physical and mental subscales with patients' sex; however, mortality after surgery and QoL was negatively associated with patients' age , which their findings were comparable to our results (6). One study from Iran on 81 patients with intertrochanteric fracture who were surgically treated and the QoL was assessed by the SF-36 tool, reported the mean QoL score of 48.5±17.7 among enrolled patients, and further adjusted analysis found statistically significant associations between patient age, economic status, the fracture to surgery interval, and the QoL (P<0.001), concluding the overall QoL of these patients being moderate to low and highlighted the importance of appropriate follow-up and psychological support, especially in older individuals with intertrochanteric fractures (15).

Studies from other centers around the world also have investigated the QoL parameters in patients with intertrochanteric fractures (14, 19, 20, 21). In a study on 86 patients aged>60 years-

old with isolated and unstable intertrochanteric fractures and were in two groups of intramedullary nailing and antirotator proximal femoral nail implant (42 cases) and cementless bipolar hemiarthroplasty (44 cases) and were followed for 24 months and QoL was assessed by various tools, the results were indicative of better social functioning and mobility scores in the internal fixation group with significantly better improvement rates in this group over time ($P<0.01$) (14). A long-term cohort study of 200 cases with intertrochanteric fracture and the healthy age- and sex-matched controls found that QoL of patients with this injury had significantly lower QoL especially regarding activities in daily living functions compared to the healthy controls (19). One investigation on 117 patients with unstable intertrochanteric fractures treated in two groups of proximal femoral nail (66 cases), hemiarthroplasty (42 cases), or total hip arthroplasty (9 cases), found the proximal femoral nailing the superior method in terms of patient mobilization and QoL parameters (20).

In an interventional trial on 482 cases of senile osteoporotic intertrochanteric fracture of femur who were treated by proximal femoral intramedullary fixation and then received zoledronic acid or calcium carbonate/Vitamin D3, the results showed that prescription of zoledronic acid postoperatively improves both bone metabolism and QoL of patients making this agent a viable option to improve the QoL after surgery and facilitate bone healing (22). One retrospective cohort study on elderly patients with intertrochanteric fractures in terms of QoL, showed age is an independent factor of achieving better outcomes that should be taken into consideration in patient management (21), which was similar to our findings.

Other comparative research on QoL on different kinds of injuries also exist. In a study by Hagino et al., it was discovered that patients with pelvic fractures, particularly intertrochanteric fractures, have a lower QoL compared to those with other types of fractures (23). The QoL in patients decreased slightly in all eight subscales of the SF-36 tool in a study by Fanian et al., with the greatest decrease in the areas of limited activity due to physical injury and physical function, which is consistent with the findings of the current study (12). Because this sort of fracture has a detrimental impact on patients in general, fractures in the elderly patient have an even greater

impact (4). The amplification of all of the aforementioned situations would be beyond comprehension due to the absence of good physical fitness and minerals in bodies of elderly population (19).

In this study, female patients had a lower QoL than male patients. Given males' better physical status of bones, and the issues created by many pregnancies and births in women, and their direct influence on physical ability, females' lower QoL in this study could be justified. Also, patients of younger ages had a greater general QoL and its subscales in this study. Factors such as chronic illness, bone resorption, inability to do personal duties, and diminished control over the environment appear to impair and reduce the QoL in older individuals (7).

This study had some limitations. The limited sample size and follow-up period was among the top limitations. Lack of records on complication rates and outcomes after surgery was another limitation. Also, the QoL was assessed only by one tool that could be expanded in future studies to achieve the best estimations on the affected individuals by intertrochanteric fractures. In contrast, this study had some strengths, too. Including various factors in study analysis was the main strength of this study to evaluate the QoL by several stratifications. Including two groups of patients with and without surgical treatment and comparing QoL was the other study strength. Findings of this study could be used in future to improve the QoL of elderly population affected by this injury and similar research in the field to benefit the patients.

Conclusion

The findings of this study revealed that patients with an intertrochanteric femoral fracture had a poor QoL in all aspects. The overall QoL was significantly higher among male patients with this kind of fracture, younger patients, those who underwent surgical treatment, and the falls from higher heights. These findings highlight the necessity of long-term follow-up in patients with intertrochanteric fractures both medically and psychosocially to improve the treatment outcomes and QoL.

Acknowledgements

The authors would like to thank all who contributed to this study.

References

1. Sun D, Wang C, Chen Y, Liu X, Zhao P, Zhang H, et al. A meta-analysis comparing intramedullary with extramedullary fixations for unstable femoral intertrochanteric fractures. Medicine (Baltimore). 2019;98(37):e17010.
2. Al-yassari G, Langstaff RJ, Jones JW, Al-Lami M. The AO/ASIF proximal femoral nail (PFN) for the treatment of unstable trochanteric femoral fracture. Injury. 2002;33(5):395-9.
3. Court-Brown CM, Heckman JD, McQueen MM, Ricci WM, Tornetta P, McKee MD. Rockwood and Green's fractures in adults. Eighth edition ed. Philadelphia: Wolters Kluwer Health; 2015.
4. Adeyemi A, Delhougne G. Incidence and Economic Burden of Intertrochanteric Fracture: A Medicare Claims Database Analysis. JB JS Open Access. 2019;4(1):e0045.
5. Bahrami M. Treatment of intertrochanteric fracture with DHS. Journal of Babol University of Medical Sciences. 2003;5(2):47-50.
6. Shafiei SH, Ghaderzadeh S, Rastegar M, Siavashi B, Golbakhsh M, Mortazavi SMJ. Mortality Rate and Quality of Life in Patients with Intertrochanteric Fractures Treated with Dynamic Hip Screw. Journal of Orthopedic and Spine Trauma. 2022;8(2).
7. Nasab SAM, Khorramdin E. The assessment of mortality and quality of life after intertrochanteric fracture of femur in patients older than 60 at Emam Khomeini Hospital of Ahvaz. Pak J Med Sci. 2017;33(4):895-8.
8. Sarrafan M, Dashtbozorg M, Mehdinassab M, Abdolhossein S, Azma M. Outcome of surgical fixation of intertrochanteric hip fracture. Iranian Journal of Orthopedic Surgery. 2020;8(1):9-13.
9. Singh D, Singh A, Singh G, Singh M, Sandhu A, Sandhu KS. Comparative Study of the Management of Intertrochanteric Fracture Femur With Proximal Femoral Nail vs. the Dynamic Hipscrew With Derotation Screw in Elderly Population. Cureus. 2021;13(11):e19431.
10. Hayat Z, Varacallo M. Surgical Management of Femoral Neck Fractures. StatPearls. Treasure Island (FL): StatPearls Publishing

Copyright © 2023, StatPearls Publishing LLC.; 2023.

11. Tidermark J. Quality of life and femoral neck fractures. Acta Orthopaedica Scandinavica. 2003;74(2):1-62.
12. FANIAN H, BOUZARI POUR BOUEINI B, SHAMANDI Z. Comparison of quality of life in patients with femoral shaft fracture, undergone surgery and normal individuds in Al-Zahra and Kashani Hospitals Isfahan. JOURNAL OF SHAHREKORD UNIVERSITY OF MEDICAL SCIENCES. 2005;7(2):9-14.
13. Amarilla-Donoso FJ, López-Espuela F, Roncero-Martín R, Leal-Hernandez O, Puerto-Parejo LM, Aliaga-Vera I, et al. Quality of life in elderly people after a hip fracture: a prospective study. Health Qual Life Outcomes. 2020;18(1):71.
14. Desteli EE, İmren Y, Erdoğan M, Aydagün Ö. Quality of Life Following Treatment of Trochanteric Fractures with Proximal Femoral Nail versus Cementless Bipolar Hemiarthroplasty in Elderly. Clin Invest Med. 2015;38(2):E63-72.
15. Fakoor M, Atshani MA, Ghasemi MA. Return to Previous Activities and Quality of Life in Patients with Femoral Intertrochanteric Fracture Surgery. Jundishapur Scientific Medical Journal. 2016;15(4):415-22.
16. Ware JE, Jr., Gandek B. Overview of the SF-36 Health Survey and the International Quality of Life Assessment (IQOLA) Project. J Clin Epidemiol. 1998;51(11):903-12.
17. Montazeri A, Goshtasebi A, Vahdaninia M, Gandek B. The Short Form Health Survey (SF-36): translation and validation study of the Iranian version. Qual Life Res. 2005;14(3):875-82.
18. Shamsi A, Ziya SS, Shahabinejad R. Trend of Quality of Life in Patients with Traumatic Hand Injury. Military Caring Sciences. 2019;5(3):165-72.

19. Willig R, Keinänen-Kiukaaniemi S, Jalovaara P. Mortality and quality of life after trochanteric hip fracture. Public Health. 2001;115(5):323-7.
20. KARADEMIR G, Bilgin Y, Demirel M, Polat G, Erşen A, KIZILKURT T, et al. Effect of Treatment Modality on Mobility and Quality of Life in Unstable Intertrochanteric Fractures. Acıbadem Üniversitesi Sağlık Bilimleri Dergisi. 2022;13(4):537-42.
21. Ju JB, Zhang PX, Jiang BG. Risk Factors for Functional Outcomes of the Elderly with Intertrochanteric Fracture: A Retrospective Cohort Study. Orthop Surg. 2019;11(4):643-52.
22. Liu Z, Li CW, Mao YF, Liu K, Liang BC, Wu LG, et al. Study on Zoledronic Acid Reducing Acute Bone Loss and Fracture Rates in Elderly Postoperative Patients with Intertrochanteric Fractures. Orthop Surg. 2019;11(3):380-5.
23. Hagino H, Nakamura T, Fujiwara S, Oeki M, Okano T, Teshima R. Sequential change in quality of life for patients with incident clinical fractures: a prospective study. Osteoporos Int. 2009;20(5):695-702.
