# Supplementary material for: Evaluation of quality of life and associated factors in patients with intertrochanteric femoral fracture
Source: PLoS One. 2023 Nov 21;18(11):e0293686. doi: 10.1371/journal.pone.0293686 (PMC10662741; doi:10.1371/journal.pone.0293686)
Supplement: Attachment — Submitted filename: Reviewer comments.docx [file pone.0293686.s002.docx]

| **Page No.** | **Line No.** | **Comment** |
| --- | --- | --- |
| 2 | 38 | “Cohort study” This is a cross sectional study and cannot be called a cohort study as there are no groups (exposed and non-exposed) which have been studied before the onset of exposure (Inter trochanteric Fracture). Hence, authors are requested to remove the word “Cohort”. |
| 4 | 94 | Same comment as above. Kindly remove the word “Cohort” |
| 4 | 98-99 | Authors can add details or references for the basis of selection of exclusion criteria such as “admission to the hospital being more than three weeks,”, “if the cause of fracture was car accident or fall from a height of more than two meters,” |
| Overall, the authors have worked well on the manuscript. However, considering the designations of the authors, they can go for higher levels of statistical analysis such as deriving of predictive models using regression analysis to assess if the factors such as age group, height of fall, gender, type of treatment opted by the patients etc. contribute significantly to the total SF 36 score or not. | | |
